# LC-MS/MS-based metabolomics approach identified novel antioxidant flavonoids associated with drought tolerance in citrus species

**DOI:** 10.3389/fpls.2023.1150854

**Published:** 2023-08-10

**Authors:** Muhammad Junaid Rao, Bihong Feng, Muhammad Husnain Ahmad, Muhammad Tahir ul Qamar, Muhammad Zeshan Aslam, Muhammad Fasih Khalid, Sajjad Hussain, Ruimin Zhong, Qurban Ali, Qiang Xu, Chongjian Ma, Lingqiang Wang

**Affiliations:** ^1^ State Key Laboratory for Conservation and Utilization of Subtropical Agro-Bioresources, Guangxi Key Laboratory of Sugarcane Biology, College of Agriculture, Guangxi University, Nanning, Guangxi, China; ^2^ Key Laboratory of Horticultural Plant Biology (Ministry of Education), Key Laboratory of Biology and Genetic Improvement of Horticultural Crops (Ministry of Agriculture), Huazhong Agricultural University, Wuhan, Hubei, China; ^3^ Integrative Omics and Molecular Modeling Laboratory, Department of Bioinformatics and Biotechnology, Government College University Faisalabad (GCUF), Faisalabad, Pakistan; ^4^ Southwest Florida Research and Education Center, Horticultural Sciences Department, Institute of Food and Agricultural Science, University of Florida, Immokalee, FL, United States; ^5^ Department of Horticulture, Faculty of Agricultural Sciences and Technology, Bahauddin Zakariya University, Multan, Pakistan; ^6^ Henry Fok School of Biology and Agriculture, Shaoguan University, Shaoguan, China; ^7^ Key Laboratory of Integrated Management of Crop Diseases and Pests, Department of Plant Pathology, College of Plant Protection, Nanjing Agricultural University, Nanjing, China

**Keywords:** citrus, drought stress, flavonoids, antioxidant activity, stress tolerance

## Abstract

Citrus fruits are cultivated around the world, and they face drought stress frequently during their growth and development. Previous studies showed that citrus plants biosynthesized flavonoid compounds in response to abiotic stress. In this study, we have quantified 37 flavonoid compounds from the leaves of three distinct citrus species including sour orange (drought-tolerant), pummelo ‘Majia you pummelo’ (drought-sensitive), and lemon (drought-sensitive). The 37 flavonoids consisted of 12 flavones, 10 flavonols, 6 flavanones, 5 isoflavanones, and 1 each for chalcone, flavanol, flavanonol, and flavone glycoside. Drought stress differentially altered the flavonoid metabolism in drought-tolerant and drought-sensitive citrus species. The kaempferol 3-neohesperidoside was 17-fold higher in sour orange (124.41 nmol/L) after 18 days of drought stress than lemon (7.33 nmol/L). In sour orange, neohesperidin (69.49 nmol/L) was 1,407- and 37-fold higher than pummelo and lemon, respectively. In sour orange, some flavonoids were significantly increased, such as vitexin, neohesperidin, cynaroside, hyperoside, genistin, kaempferol 3-neohesperidoside, eriocitrin, and luteolin, in response to drought stress, whereas in lemon, these flavonoids were significantly decreased or not altered significantly in response to drought stress. Moreover, the total contents of flavonoids and antioxidant activity were increased in sour orange as compared with pummelo and lemon. The genes associated with flavonoid biosynthesis (*PAL*, *CHI*, *FLS*, *GT1*, *F3H*, *F3’M*, *C4H*, *4CL*, *FLS*, *FG2*, *FG3*, and *CYP81E1*) were more highly expressed in sour orange leaves than in pummelo and lemon after drought stress. These outcomes showed that pummelo and lemon failed to biosynthesize antioxidant flavonoids to cope with the prolonged drought stress, whereas the sour orange biosynthesized fortified flavonoid compounds with increased antioxidant activity to detoxify the harmful effects of reactive oxygen species produced during drought stress.

## 1 Introduction

Citrus is one of the most important fruit crops, and it is cultivated around subtropical and tropical regions (>140 countries) of the world (Liu et al., 2012). China, Brazil, the United States of America, and India are major citrus-producing countries (Liu et al., 2012). Commercially cultivated citrus fruits include pummelos, oranges, lemons, limes and mandarins (Rao et al., 2021b). Most of the cultivated and wild citrus species are native to China such as wild mandarins and pummelos (Rao et al., 2021b). Horticultural crops including citrus species are facing many abiotic and biotic stresses that restrict citrus production by 50%–100% (Liu et al., 2012; Malik et al., 2015; Hussain et al., 2018; Rao et al., 2018; Francini and Sebastiani, 2019).

Drought stress is one of the major abiotic stress factors that affect plant growth and crop productivity (Bhardwaj et al., 2015). Drought stress enhances the metabolic process, disrupts the electron transport system, and disturbs the normal homeostasis of a plant cell, which increases the production of reactive oxygen species (ROS) (Hussain et al., 2018; Rao et al., 2020). Overproduction of ROS directly affects the photosynthesis apparatus; reacts with nucleic acids, DNA, cell membrane, and proteins; inhibits metabolic enzyme activities; and eventually causes cell death (Hussain et al., 2018; Rao et al., 2021a). The citrus cultivated in semiarid regions is more prone to drought stress. Severe drought stress caused heavy fruit drops and increased the cracking and folding of citrus fruit, eventually causing a 70% decline in yield (Francini and Sebastiani, 2019). Cultivated citrus species such as sweet oranges, pummelos, and lemons are more sensitive to stress (Rao et al., 2018). Some tolerant citrus species or rootstocks are recommended to overcome the drought stress in semiarid areas of citrus.

Some plants, including citrus fruits, produce many metabolites for their normal defense, growth, and development functioning (Kabera et al., 2014; Rao et al., 2021a). Previous studies suggested that the cultivated citrus species enhances the biosynthesis of secondary metabolites when exposed to biotic and abiotic stresses (Zandalinas et al., 2017a; Zandalinas et al., 2017b; Dala Paula et al., 2018). Citrus leaves accumulate high levels of flavonoids to cope with the negative effects of water-deficit conditions (Zandalinas et al., 2017b; Rao et al., 2020). Drought-tolerant citrus rootstocks such as sour orange and *Poncirus trifoliata* have high concentrations of metabolites, whereas the cultivated pummelos and lemons are sensitive to drought stress, and these species have fewer total metabolites (Naser and Mohammad, 2016; Zandalinas et al., 2017a; Rao et al., 2018; Rao et al., 2021a). Phenolic compounds are recognized for their high antioxidant activity and functions in signaling, reproduction, antimicrobial activity, and regulation of plant cell physiology (Kabera et al., 2014; Mathesius, 2018; Rao et al., 2021a).

Flavonoids are a representative group of secondary metabolites that have been reported to play a role in protecting cellular organelles by detoxifying the ROS produced during abiotic and biotic stress in many plants (Scalbert et al., 2005; Pourcel et al., 2007; Borges Bubols et al., 2013). Citrus leaves produce diverse secondary metabolites that provide a secondary defense by detoxifying the ROS produced during different abiotic and biotic stresses (Zandalinas et al., 2017b). Recently, it was reported that citrus species that have a high level of flavonoids or rapidly biosynthesized flavonoids after drought stress can acclimatize to stress condition more efficiently than those species who have fewer metabolites or stimulate late biosynthesis of flavonoids (Rao et al., 2021a). However, in the past decade, most research has focused on the nutritional profiling of cultivated citrus species; flavonoid profiling of leaves in response to drought stress is less studied in citrus species, and whether flavonoids effectively scavenge ROS remains to be determined.

In this study, we selected three citrus species, namely, sour orange (drought-tolerant), pummelo (known as ‘Majia you pummelo’) (drought-sensitive), and lemon (drought-sensitive), to quantify different classes of flavonoids in response to drought stress. In addition, we performed antioxidant activity and capacity and developed a flavonoid biosynthesis pathway that was activated in response to drought stress in sour oranges. We evaluated some physiological and biochemical parameters at different time points of drought stress in three citrus species. This research expands our current understanding of specialized individual flavonoid compounds that are correlated with drought in citrus species and provides information for their further drought resistance research and variety breeding.

## 2 Materials and methods

The three citrus species, sour orange (*Citrus aurantium*), pummelo (*Citrus maxima*), and lemon (*Citrus limon*) were grown under controlled conditions (26°C, 60% humidity, and 10,000 LUX light intensity) at the College of Agriculture, Guangxi University, Nanning, China. All seeds of these species were planted on seed beds separately on 15 November 2021 and transplanted as seedlings into big pots on 10 January 2022. After 5 months, the water was stopped for 18 days, and leaves were harvested at control (well-watered), 6, 12, and 18 days of drought stress (DDS). According to the leaves’ symptoms, we have selected three time points for flavonoid quantification (control, 12DDS, and 18DDS) and four time points for physiological and biochemical parameters. At each time point, the control and stressed leaves samples were collected from each citrus species with three biological repeats and immediately frozen into liquid nitrogen followed by vacuum freeze-drying and stored until use. For each biological replicate, six leaves were harvested.

### 2.1 Flavonoid profiling

Flavonoids profiling of citrus samples was performed by ultra-performance liquid chromatography-tandem mass spectrometry (UPLC-MS/MS). Freeze-dried citrus leaves were grounded into powder in liquid nitrogen, and 50 mg of leaf powder was used for flavonoids qualitative and quantitative analysis. Flavonoids were extracted by adding 500 μl of 70% aqueous methanol to each grounded citrus sample and 10 μl of internal standard “Rutin” (4,000 nmol/L). The ultrasonic extraction (Ultrasonic Model: KQ5200E, Kunshan Ultrasonic Instrument Co., Ltd. with 80% power and 5°C) was performed for 30 min followed by centrifugation for 5 min at 12,000 rpm, and finally, the supernatant was collected. A microporous membrane of 0.22 μm was used to filter the supernatant, and the filtrate was stored for further analysis. UPLC-MS/MS analysis and multiple reactions monitoring (operated in both −4,500 negative ion mode and 5,500 positive ion mode) were implemented as defined before (Chen et al., 2013; Rao et al., 2022). The UPLC and electrospray ionization-mass spectrometry (ESI-MS/MS) conditions are represented in **Supplementary File 1**. Analyst 1.6.3 software was used for MS data analysis. The qualitatively and quantitatively mass spectrum peak was detected according to the calibration curves and retention times (RT) of the standards (**Table 1**; **Supplementary Table S1**). Multiple reactions monitoring (MRM) transition details are presented in **Supplementary Table S2**. Flavonoids qualitative analysis, quantification formula, standard equation (the calibration curve), coefficient of correlation value, and minimum and maximum detection limits of (individual flavonoid compounds) the system are represented in the **Supplementary File** (**Supplementary Table S1**).

**Table 1:** Quantified flavonoid compounds from the leaves of three citrus species.

Serial No.	Compounds	Class	Formula	Molecular weight	Ionization model	Q1 (Da)	Q3 (Da)	Retention time
1	Naringenin chalcone	Chalcones	C_15_H_12_O_5_	272.07	[M−H]−	271.1	151	4.98
2	(−)-Epigallocatechin	Flavanols	C_15_H_14_O_7_	306.07	[M+H]+	307.1	139	2.01
3	Neohesperidin	Flavanones	C_28_H_34_O_15_	610.19	[M−H]−	609.2	301.1	4.1
4	Isosakuranetin	Flavanones	C_16_H_14_O_5_	286.08	[M−H]−	285.1	164	7.12
5	Hesperetin	Flavanones	C_16_H_14_O_6_	302.08	[M−H]−	301.1	164	5.29
6	Pinocembrin	Flavanones	C_15_H_12_O_4_	256.07	[M−H]−	255.1	213.1	7.19
7	Eriocitrin	Flavanones	C_27_H_32_O_15_	596.17	[M−H]−	595.2	287.1	2.74
8	Eriodictyol	Flavanones	C_15_H_12_O_6_	288.06	[M−H]−	287.1	135	4.18
9	Taxifolin	Flavanonols	C_15_H_12_O_7_	304.06	[M−H]−	303.1	125	3.15
10	Vitexin	Flavone glycosides	C_21_H_20_O_10_	432.10	[M−H]−	431.1	311.1	2.81
11	Luteolin	Flavones	C_15_H_10_O_6_	286.05	[M−H]−	285	133	4.22
12	Sakuranetin	Flavones	C_16_H_14_O_5_	286.08	[M−H]−	285.1	165	7.06
13	Diosmin	Flavones	C_28_H_32_O_15_	608.17	[M−H]−	607.2	299.1	3.08
14	Apigenin	Flavones	C_15_H_10_O_5_	270.05	[M−H]−	269.1	117	4.98
15	Galangin	Flavones	C_15_H_10_O_5_	270.05	[M−H]−	269	211	7.26
16	Narcissin	Flavones	C_28_H_32_O_16_	624.17	[M−H]−	623.2	315.1	2.97
17	Tricetin	Flavones	C_15_H_10_O_7_	302.04	[M−H]−	301	149	3.55
18	Cynaroside	Flavones	C_21_H_20_O_11_	448.10	[M−H]−	447.1	285	2.88
19	Acacetin	Flavones	C_16_H_12_O_5_	284.07	[M−H]−	283.1	268	7.08
20	Scutellarein	Flavones	C_15_H_10_O_6_	286.05	[M−H]−	285	117	3.75
21	Limocitrin	Flavones	C_17_H_14_O_8_	346.07	[M−H]−	345.06	315.02	3.82
22	Chrysin	Flavones	C_15_H_10_O_4_	254.06	[M−H]−	253.1	143.1	7
23	Baimaside	Flavonols	C_27_H_30_O_17_	626.15	[M−H]−	625.1	300	2.51
24	Astragalin	Flavonols	C_21_H_20_O_11_	448.10	[M−H]−	447.1	284	3.09
25	3,7-Di-O-methylquercetin	Flavonols	C_17_H_14_O_7_	330.07	[M]−H]−	329.1	314.1	6.51
26	Rutin	Flavonols	C_27_H_30_O_16_	610.15	[M−H]−	609.1	300	2.74
27	Quercitrin	Flavonols	C_21_H_20_O_11_	448.10	[M−H]−	447.1	300	3.14
28	Kaempferide	Flavonols	C_16_H_12_O_6_	300.06	[M−H]−	299.1	284	7.33
29	Kaempferol 3-neohesperidoside	Flavonols	C_27_H_30_O_15_	594.16	[M−H]−	593.2	284	2.8
30	Laricitrin	Flavonols	C_16_H_12_O_8_	332.05	[M−H]−	331.1	316	4.28
31	Myricetin	Flavonols	C_15_H_10_O_8_	318.04	[M−H]−	317	151	3.5
32	Hyperoside	Flavonols	C_21_H_20_O_12_	464.09	[M+H]+	465.1	314.9	2.84
33	2′-Hydroxygenistein	Isoflavanones	C_15_H_10_O_6_	286.05	[M−H]−	285	217.1	4.07
34	2′-Hydroxydaidzein	Isoflavanones	C_15_H_10_O_5_	270.05	[M−H]−	269	225.1	3.54
35	Genistin	Isoflavanones	C_21_H_20_O_10_	432.11	[M−H]−	431.1	268	3
36	Formononetin	Isoflavanones	C_16_H_12_O_4_	268.07	[M−H]−	267.1	252.1	5.98
37	Calycosin	Isoflavanones	C_16_H_12_O_5_	284.07	[M−H]−	283.1	268	4.31

Q1, parent ion; Q3, daughter ion.

### 2.2 RNA isolation and quantitative RT-PCR

RNA extraction kit Trizol (Takara) was used to extract the total RNA from 100 mg of citrus leaves. The complementary DNA (cDNA) was synthesized with the QRT-reverse transcriptase kit (Vazyme, R223-01) from drought-stressed citrus leaf samples. One microgram of total RNA has been used for the synthesis of cDNA. The gDNA wiper mix was used to remove genomic DNA from each sample. A quantitative real-time polymerase chain reaction (qRT-PCR) light cycler 480 II instrument (Roche) was used, whereas the citrus β-actin gene was used as an internal reference. The qPCR primers of 15 genes are characterized in the supplementary primer table.

### 2.3 Extraction procedure of total phenolics and total flavonoid contents

#### 2.3.1 Extraction

For total phenolic and total flavonoid contents, 100 mg of citrus leaf samples was homogenized in 5 ml of methanol 80% (Velioglu et al., 1998). After that, the samples were incubated at room temperature for 2 h on an orbital shaker at 200 rpm. After shaking, the citrus leaf samples were centrifuged for 10 min at 10,000 rpm, and the supernatant was collected for further analysis. This extraction procedure has been repeated twice, and both supernatants were combined in one tube for the estimation of the total contents of phenolics and flavonoids.

#### 2.3.2 Total phenolic contents

The Folin–Ciocalteu reagent (FCR) methodology was used to calculate the total phenolic contents (Velioglu et al., 1998). The 300 μl of the above-prepared methanolic extract was taken in a fresh 10-ml tube and mixed with 2.25 ml of 10-fold diluted FCR solution. After 6 min incubation at room temperature, the sodium carbonate (Na_2_CO_3_) (60 g/L) solution of 2.25 ml was added, and the reaction mixture was incubated at room temperature for 2 h. Then, absorbance of each sample was measured at 725 nm on a UV-1800 (model Shimadzu, Japan) spectrophotometer. The gallic acid standard curve was generated, and the results were quantified in milligrams of gallic acid-equivalents (GAE)/gram of citrus leaves samples (mg GAE/g).

#### 2.3.3 Estimation of total flavonoid content

For total flavonoids estimation, zero-point 5 ml of the above-prepared citrus leaves solution was taken in a fresh tube followed by the addition of 2.25 ml of distilled water. Then, after gentle inversion, 0.15 ml of sodium nitrite (NaNO_2_) 5% solution was added and incubated for 6 min followed by the addition of 0.3 ml of aluminum chloride hexahydrate 10% solution (AlCl_3_·6H_2_O) and vertex, followed by incubation at room temperature for 5 min. Then, 1M concentration of sodium hydroxide (NaOH) was prepared, and 1 ml of this solution was added to the above reaction mixture and mixed well by gentle inversions for 2 min. The absorbance reading was taken at 510 nm on the UV-1800 (Shimadzu, Japan) spectrophotometer (Dewanto et al., 2002). The Rutin standard curve was produced to determine the total flavonoid content in milligrams of rutin equivalents (RE) of citrus leaf samples (mg RE/g).

### 2.4 Hydrogen peroxide determination

H_2_O_2_ level was measured by taking a hundred milligrams of citrus leaves tissues and 1 ml of trichloro-acetic acid (1%) was added followed by a 10-min ice bath as described earlier (Velikova et al., 2000). The absorbance of each sample was taken at 390 nm by using the UV-1800 (Shimadzu, Japan) spectrophotometer, and the hydrogen peroxide level was calculated in micromoles/gram by generating a standard curve using industrial H_2_O_2_.

### 2.5 DPPH free radical scavenging assay

From the citrus leaf powder, the antioxidant activity and capacity were measured by using the DPPH (2,2-diphenyl-1-picrylhydrazyl) technique as discussed previously (Ozgen et al., 2010). A total of 100 mg of citrus leaf samples had been homogenized in 1 ml of extraction solution (ethanol 70%, water 29%, and acetic acid 1%). After that, the leaves sample was centrifuged at 10,000 rpm for 8 min at room temperature. The supernatant (0.03 ml) were collected in a fresh tube followed by the addition of DPPH (0.1 mM) 2.97 ml in each sample accompanied by incubation in the dark, for 30 min, at room temperature. The absorbance reading was taken at 517 nm on the UV-1800 (Shimadzu, Japan) spectrophotometer. The different concentrations of Trolox were used as a standard, and the standard curve was generated to calculate the antioxidant capacity described in millimolar of Trolox/100 mg. Moreover, the antioxidant activity (free radical scavenging %) was determined by using the following formula:


Antioxidantactivity(%)=100×[1−{sample OD/control OD}]

### 2.6 Chlorophyll and relative water contents

Chlorophyll a and b contents were measured by taking 500 mg of citrus leaf powder followed by the addition of 10 ml of acetone 80% (v/v) (Sumanta et al., 2014). After gentle inversion for 3 min, the reaction solution was incubated at room temperature (in the dark) for 4 h followed by centrifugation at 10,000 rpm for 10 min. After that, the supernatant was collected in a new tube, and for the remaining tissues, the same procedure was repeated, and the supernatant was added to the new tube. The absorbance of the supernatant was taken at 645 and 663 nm by using the UV-1800 (model Shimadzu, Japan) spectrophotometer. The chlorophyll a and b contents were determined in milligrams per liter (mg/L) by putting the absorbance values in the following formula, while pure acetone was used as a blank:


Chlorophyll a=(12.7×OD663)−(2.69×OD645)

Chlorophyll b=(22.9×OD645)−(4.68×OD663)

Relative water content (RWC) was calculated by taking the fresh leave weight, dry leave weight, and turgid leave weight, and then, the following formula was used for RWC:


RWC(%)=[(freshweight−dryweight)/(turgidweight−dryweight)]×100

### 2.7 Statistical analysis

Statistical analysis was performed by using Statistix 8.1 statistical software (Tallahassee, FL, USA). Each bar in the graphs signifies the mean value of three biological repeats. The individual flavonoid compounds, physiological and biochemical parameter graphs, and standard error were performed by using Excel (Microsoft Corp., Redmond, WA, USA) program. A multi-statistical analysis method was implemented for cluster analysis by using the drought-stressed flavonoid data to characterize all the individual compounds with heterogeneity as high as possible between categories and the highest possible homogeneity in the same category. The relative abundance of each flavonoid compound was used and normalized by using R software (https://www.r-project.org/, accessed on 22 July 2022) to perform the principal component analysis (PCA) and hierarchical clusters analysis (HCA). Venn interactive flower plot and network analysis of flavonoid compounds were performed by using the free online program, the EVenn (http://www.ehbio.com/test/venn/#/, accessed on 14 September 2022).

## 3 Results

### 3.1 Drought stress phenotype on the leaves of citrus species

**Figure 1:**
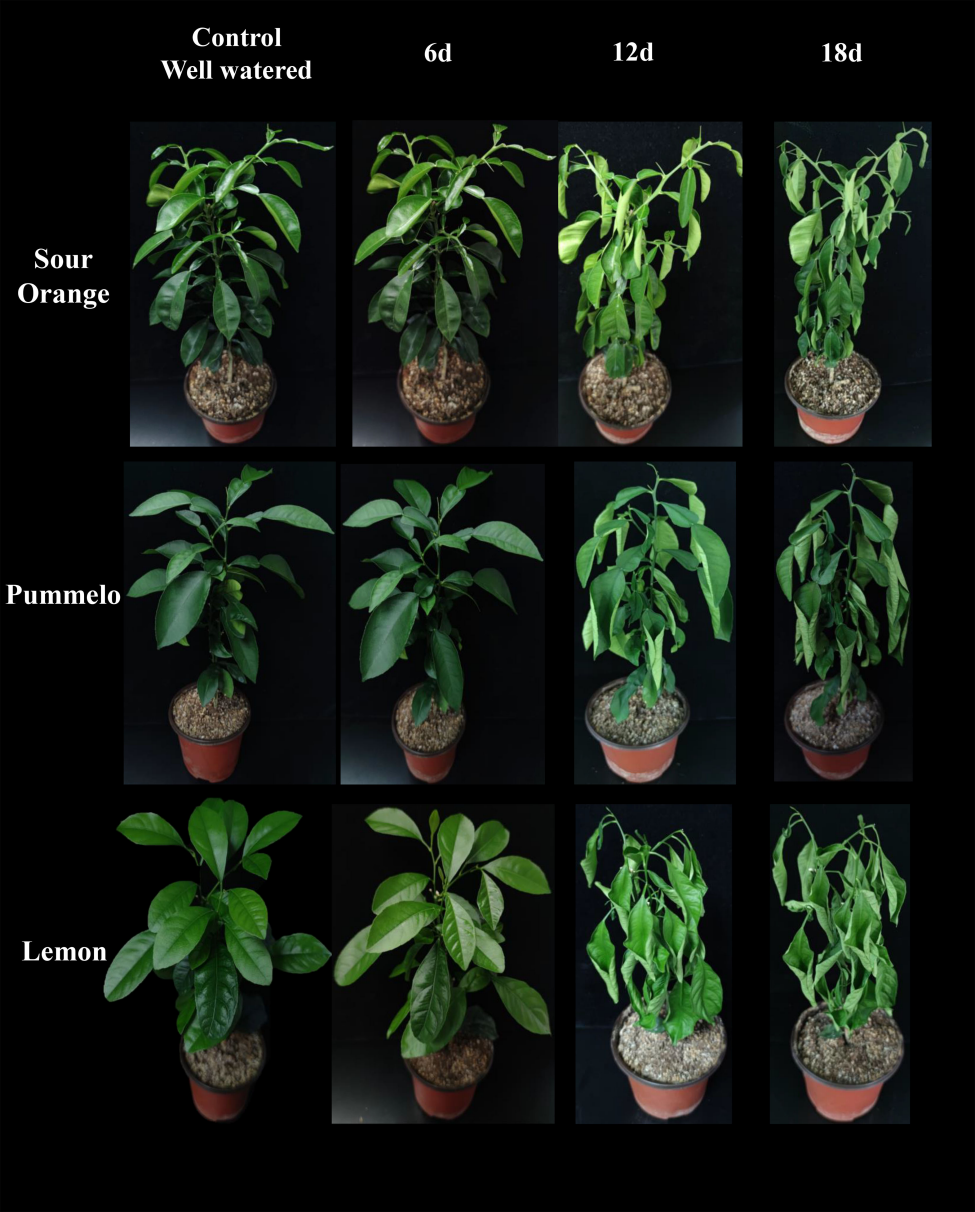
Drought stress effect on the leaves of three citrus species. 6d, 6 days of drought stress; 12d, 12 days of drought stress; 18d, 18 days of drought stress.

The phenotype of drought stress on sour orange, pummelo, and lemon leaves was represented (**Figure 1**). After 12 and 18 days of drought stress, significant drought symptoms were observed on the leaves of citrus species, so we harvested leaves at control, 12DDS, and 18DDS for the flavonoids study (**Figure 1**). A total of 37 flavonoids were quantified from the leaves of three citrus species (**Table 1**; **Supplementary Table S1**). These flavonoid compounds include 12 flavones, 10 flavonols, 6 flavanones, 5 isoflavanones, and 1 each for flavanol, flavone glycoside, chalcone, and flavanonol (**Table 1**).

### 3.2 Clustering and grouping of flavonoids and citrus species under drought stress

**Figure 2:**
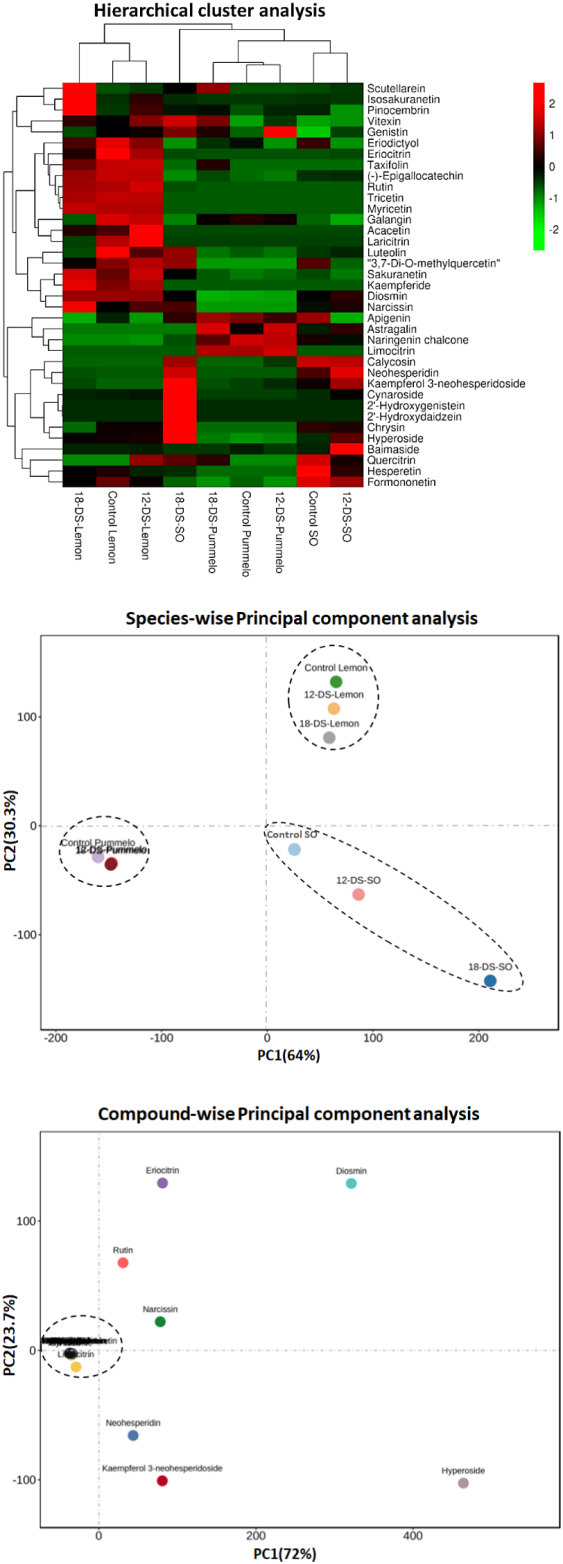
Hierarchical cluster analysis and principal component analysis of flavonoid compounds of citrus leaves under drought stress. Heatmap row denotes the individual flavonoid compounds (rows were normalized). Abbreviations: SO, sour orange; 12-DS, 12 days of drought stress, 18-DS, 18 days of drought stress.

Hierarchical clustering analysis (HCA) was performed among citrus species and individually quantified flavonoid compounds (**Figure 2**). Different drought intervals of citrus species made two major (lemon cluster and second pummelo, sour orange cluster) and four sub-clusters on HCA (**Figure 2**). The control and 12DDS lemon variety made the same cluster, whereas after 18DDS, the lemon variety made a slightly different but close to the control and 12DDS lemon cluster (**Figure 2**). A similar grouping was observed in the pummelo (**Figure 2**). Sour orange showed distinct behavior from lemon and pummelo species; after 18DDS, the sour orange made a unique cluster on HCA, whereas the control and 12DDS sour orange groups made a separate group (**Figure 2**). The eight flavonoid compounds, cynaroside, 2′-hydroxygenistein, 2′-hydroxydaidzein, calycosin, chrysin, neohesperidin, kaempferol 3-neohesperidoside, and vitexin, were uniquely higher in the leaves of sour orange under 18DDS, whereas these compounds were significantly lower in the pummelo and lemon drought stressed leaves (**Figure 2**). Additionally, the control and 12DDS sour orange group have a high concentration of baimaside, hyperoside, and quercitrin compounds than the pummelo and lemon groups (**Figure 2**). The high concentration of aforementioned flavonoids in drought-tolerant SO and the low concentration of these flavonoids in drought-susceptible lemons revealed that these compounds play a significant role in drought tolerance of the sour orange variety.

PCAs of different citrus species (with different drought intervals) and individual flavonoid compounds were performed (**Figure 2**). Citrus variety-wise two-dimensional PCAs showed that sour orange, pummelo, and lemon made three distinct clusters (**Figure 2**). The sour orange cluster differed greatly from the pummelo and lemon clusters and showed unique characteristics on PCA (**Figure 2**). Variety-wise PCA differed greatly the highest absolute score values of PC1 (64%) on the x-axis and PC2 (30.3%) on the y-axis (**Figure 2**). In compound-wise PCA, the flavonoids were clustered near the intersection point of the x- and y-axes of the scatter plot except for hyperoside, kaempferol 3-neohesperidoside, neohesperidin, rutin, diosmin, eriocitrin, and narcissin (**Figure 2**). These seven flavonoids were scattered in compound-wise PCA and showed the highest absolute score values on PC1 (x-axis) of 72%, and PC2 (y-axis) accounted for 23.7% of the variation (**Figure 2**). PCA analysis revealed that the aforementioned seven flavonoids were uniquely altered during drought stress in citrus species; additionally, the 12- and 18DDS sour orange variety also showed distinct behavior compared to the rest of the drought-stressed citrus species in species-wise PCA (**Figure 2**).

### 3.3 Relative abundance of key flavonoids under drought stress in citrus species

**Figure 3:**
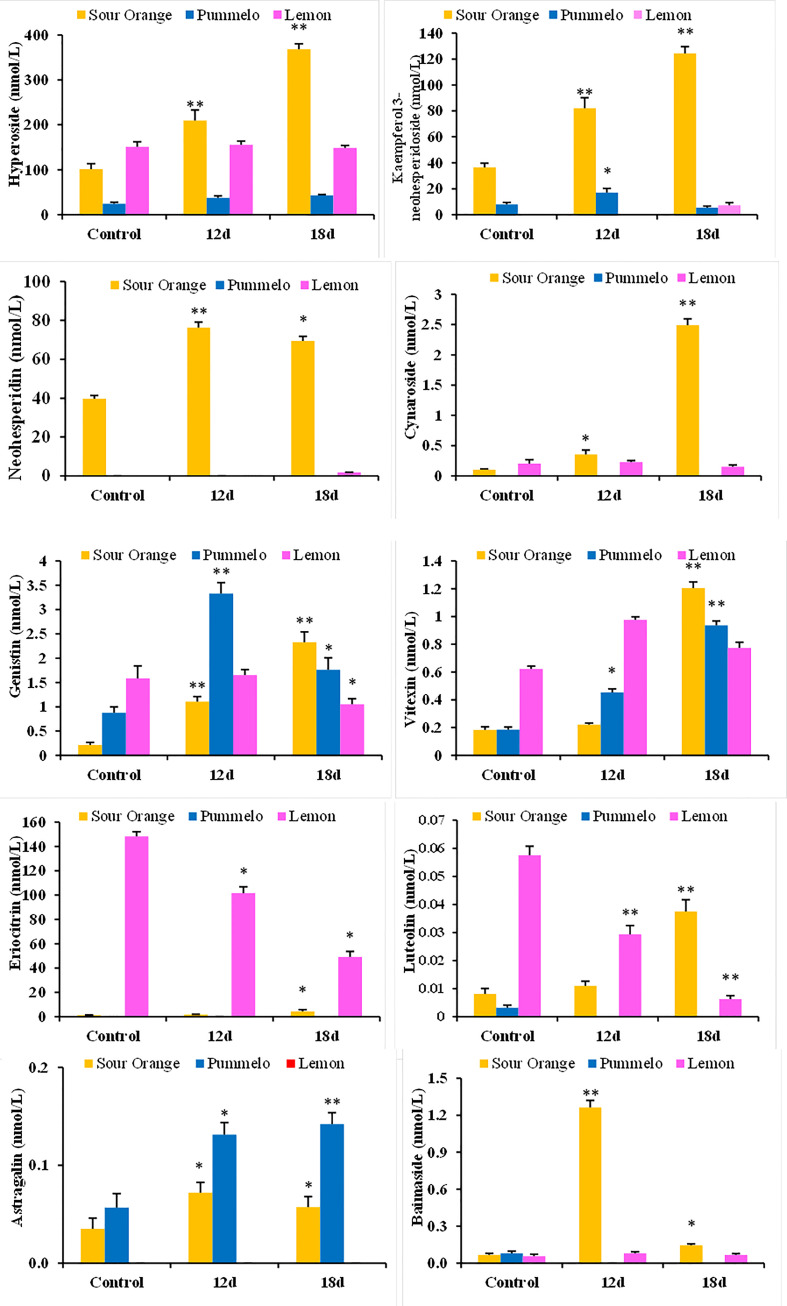
Relative abundance of flavonoid compounds (nmol/L) significantly altered under drought stress in citrus leaves. 12d, 12 days of drought stress; 18d, 18 days of drought stress. Student’s t-test was used to compare the drought-stress citrus species with their control at *p < 0.05 and **p < 0.01.

The relative abundance of the individual flavonoid has been evaluated in drought-stress citrus species (**Figure 3**). The kaempferol 3-neohesperidoside significantly increased after 12DDS and 18DDS (81.94 and 124.41 nmol/L, respectively) compared to the control (36.40 nmol/L) sour orange, whereas in lemon, it was only quantified after 18DDS (7.33 nmol/L) (**Figure 3**). Similarly, cynaroside, neohesperidin, and vitexin also increased significantly in sour orange after 12DDS and 18DDS, while in lemon, no significant change was observed after drought stress (**Figure 3**). The highest concentration of hyperoside was observed in 12DDS and 18DDS SO leaves 209.4 and 368 nmol/L, respectively, much higher than control SO (101.30 nmol/L), whereas no significant difference was observed in drought-stressed pummelo and lemon plants (**Figure 3**). Interestingly, an increasing trend of hyperoside, neohesperidin, kaempferol 3-neohesperidoside, genistin, eriocitrin, and luteolin flavonoids was observed in sour orange after 12DDS and 18DDS, whereas the opposite trend was observed in lemon plants (**Figure 3**). As the intensity of drought stress increases, the concentration of genistin, eriocitrin, and luteolin flavonoids decreased significantly in 12DDS and 18DDS lemon leaves (**Figure 3**). These outcomes showed that a high concentration and increasing trend of specific flavonoids under drought stress would assist sour orange to tolerate the prolonged drought stress, whereas the low concentration of flavonoids and decreasing trend of specific aforementioned flavonoids in response to drought stress are probably related to lemon drought susceptibility.

### 3.4 Flavonoids that significantly altered more than 1.5-fold under drought stress

After 12DDS and 18DDS, cynaroside increased by 3.44- and 24.20-fold in SO; however, lemon cynaroside decreased by 1.33-fold, after 18DDS (**Table 2**). Luteolin had a 4.62-fold increase in SO, while lemon luteolin had a 9.15-fold decrease, after 18DDS (**Table 2**). Similarly, after 18DDS, the eriocitrin level was 3.57-fold up in SO, whereas it was 2.43- and 3.02-fold down in pummelo and lemon, respectively (**Table 2**). Hyperoside (2.06 and 3.63), genistin (5.22 and 10.95), scutellarein (2.22 and 8.45), kaempferol 3-neohesperidoside (2.25 and 3.42), neohesperidin (1.92 and 1.75), and baimaside (18.89 and 2.17) were folds increased in SO after 12DDS and 18DDS, respectively; interestingly, these flavonoids were decreased or not significantly changed in lemon leaves, after 12DDS and 18DDS (**Table 2**). Astragalin increased in SO (2.05 and 1.63) and pummelo (2.31 and 2.50) after 12DDS and 18DDS, whereas in lemon, astragalin was not found (**Table 2**). In drought-tolerant sour orange leaves, specialized flavonoids such as hyperoside, genistin, eriocitrin, luteolin, cynaroside, scutellarein, kaempferol 3-neohesperidoside, and astragalin were significantly increased more than 1.5-fold after drought stress, while these flavonoids were decreased or showed no significant difference in lemon under drought stress.

**Table 2:** Flavonoid compounds fold up and fold down, after 12 and 18 days in sour orange, pummelo, and lemon leaves than their corresponding control.

Serial No.	Compounds	12d-SO Fold UP	18d-SO Fold UP	12d-P- Fold UP	18d-P- Fold UP	12d-L- Fold UP		18d-L- Fold UP
1	Cynaroside	**3.44**	**24.20**					
2	Genistin	**5.22**	**10.95**	**3.80**	**2.01**			
3	Isosakuranetin	**5.34**	**9.12**			**72.0**		**276.7**
4	Scutellarein	**2.22**	**8.45**					
5	Vitexin	1.20	**6.57**	**2.46**	**5.06**	**1.57**		
6	Luteolin	1.36	**4.62**					
7	Hyperoside	**2.06**	**3.63**	**1.53**	**1.74**			
8	Eriocitrin	1.44	**3.57**	**1.59**				
9	Kaempferol 3-neohesperidoside	**2.25**	**3.42**	**2.14**				
10	Chrysin	**2.14**	**2.72**					
11	Sakuranetin	**1.57**	**2.68**			**1.5**		**1.55**
12	Baimaside	**18.89**	**2.17**			1.42		1.20
13	Neohesperidin	**1.92**	**1.75**					
14	Astragalin	**2.05**	**1.63**	**2.31**	**2.50**			
15	Narcissin					**1.52**		**2.82**
16	Pinocembrin			**1.75**	**2.04**	**2.62**		**6.00**
17	Kaempferide					1.21		**1.56**
18	Hesperetin	**2.76**	**7.36**			**2.01**		1.23
19	Apigenin	**3.70**	1.29			**1.74**		**2.00**
20	Formononetin	1.25	**8.50**			**1.51**		**1.70**
21	Narcissin	**3.54**						
22	Neohesperidin				**1.69**			
23	Kaempferol 3-neohesperidoside				**1.5**			
24	Luteolin					**1.96**		**9.15**
25	Eriocitrin				**2.43**	1.46		**3.02**
26	Genistin							**1.50**

SO, sour orange; P, pummelo; L, lemon; 12d, 12 days of drought stress; 18d, 18 days of drought stress. Bold letters mean more than 1.5-fold changed.

After 12DDS and 18DDS, neohesperidin was 696- and 1,407.43-fold higher in SO than in pummelo (**Table 3**). Similarly, neohesperidin, kaempferol 3-neohesperidoside, and cynaroside were 37.6-, 16.96-, and 16.32-fold higher in SO than in lemon leaves, after 18DDS (**Table 3**). These results showed that the aforementioned flavonoids have some significant roles in SO drought tolerance because these flavonoids showed an increasing trend in SO, in response to drought stress, whereas in drought-susceptible lemon, these flavonoids showed a decreasing trend in response to drought stress.

**Table 3:** Showing the compounds that were more than twofold up after 12DDS and 18DDS in sour orange than 12DDS and 18DDS pummelo and lemon.

Serial No.	Compounds	12d-SO Fold up than 12d-P	18d-SO Fold up than 18d-P	12d-SO Fold up than 12d-L	18d-SO Fold up than 18d-L
**1**	Naringenin chalcone			23.43	5.47
**2**	Neohesperidin	696.04	1,407.43		37.60
**3**	Hesperetin			2.22	
**4**	Eriocitrin	15.90	151.61		
**5**	Luteolin				5.96
**6**	Sakuranetin		4.21		
**7**	Diosmin	9.23	17.09		
**8**	Apigenin				2.72
**9**	Cynaroside				16.32
**10**	Baimaside			15.85	
**11**	Rutin	8.99	28.31		
**12**	Kaempferol 3-neohesperidoside	4.82	23.34		16.96
**13**	Hyperoside	5.59	8.62		2.48
**14**	Genistin				2.21
**15**	Calycosin	7.52			

12d, 12 days of drought stress; 18d, 18 days of drought stress; SO, sour orange; P, pummelo; L, lemon.

### 3.5 Venn analysis of flavonoids under drought stress

**Figure 4:**
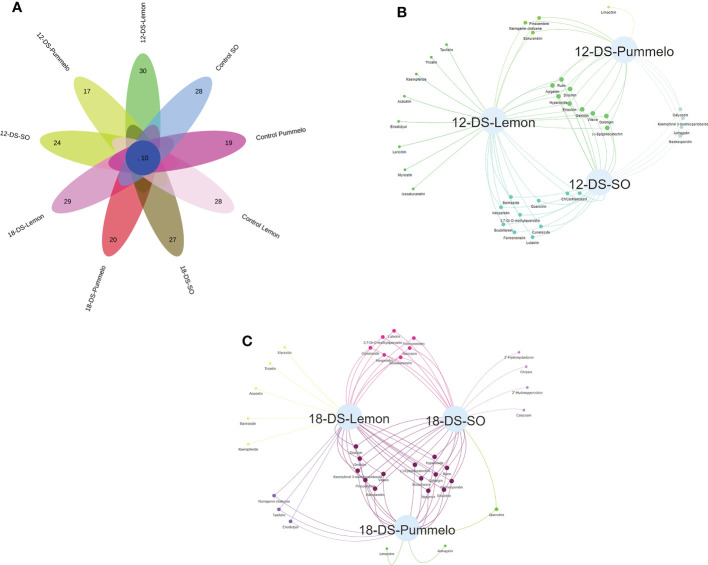
Venn analysis of flavonoids from the leaves of three citrus species. **(A)** Interactive Venn flower plot highlighting the flavonoid compounds in different citrus species under drought stress; **(B)** characterizes the Venn network analysis of three citrus species under (12-DS) 12 days of drought stress; **(C)** characterizes the Venn network analysis of three citrus species under (18-DS) 18 days of drought stress.

Among 37 quantified flavonoid compounds, the control SO and control lemon have 28 compounds, whereas the control pummelo has 19 flavonoids (**Figure 4A**). The Venn interactive flower plot showed that after 12DDS SO, lemon, and pummelo have 24, 30, and 17 compounds, respectively (**Figure 4A**). Venn network analysis revealed that nine compounds were commonly found among three citrus species after 12DDS (**Figure 4B**), whereas calycosin, kaempferol 3-neohesperidoside, neohesperidin, and astragalin were commonly found in pummelo and SO (**Figure 4B**). Myricetin, tricetin, acacetin, baimaside, and kaempferide were only observed in 18DDS lemon leaves, whereas limocitrin and astragalin were found in 18DDS pummelo leaves (**Figure 4C**). After 18DDS, 14 flavonoids were commonly found in three citrus species; however, four flavonoid compounds such as calycosin, chrysin, 2′-hydroxygenistein, and 2′-hydroxydaidzein were only quantified in the leaves of SO under 18DDS (**Figure 4C**). After 18DDS, chrysin was only found in SO, and it had a 2.72-fold higher increase than in SO control, while chrysin was not detected in pummelo and lemon under 18DDS (**Figure 4C**; **Table 2**). The presence of aforementioned compounds in drought-tolerant SO after drought stress may have some roles in protecting the cellular organelles from the harmful effects of reactive oxygen species produced during drought stress.

### 3.6 Gene expression analysis and flavonoids biosynthesis pathway

**Figure 5:**
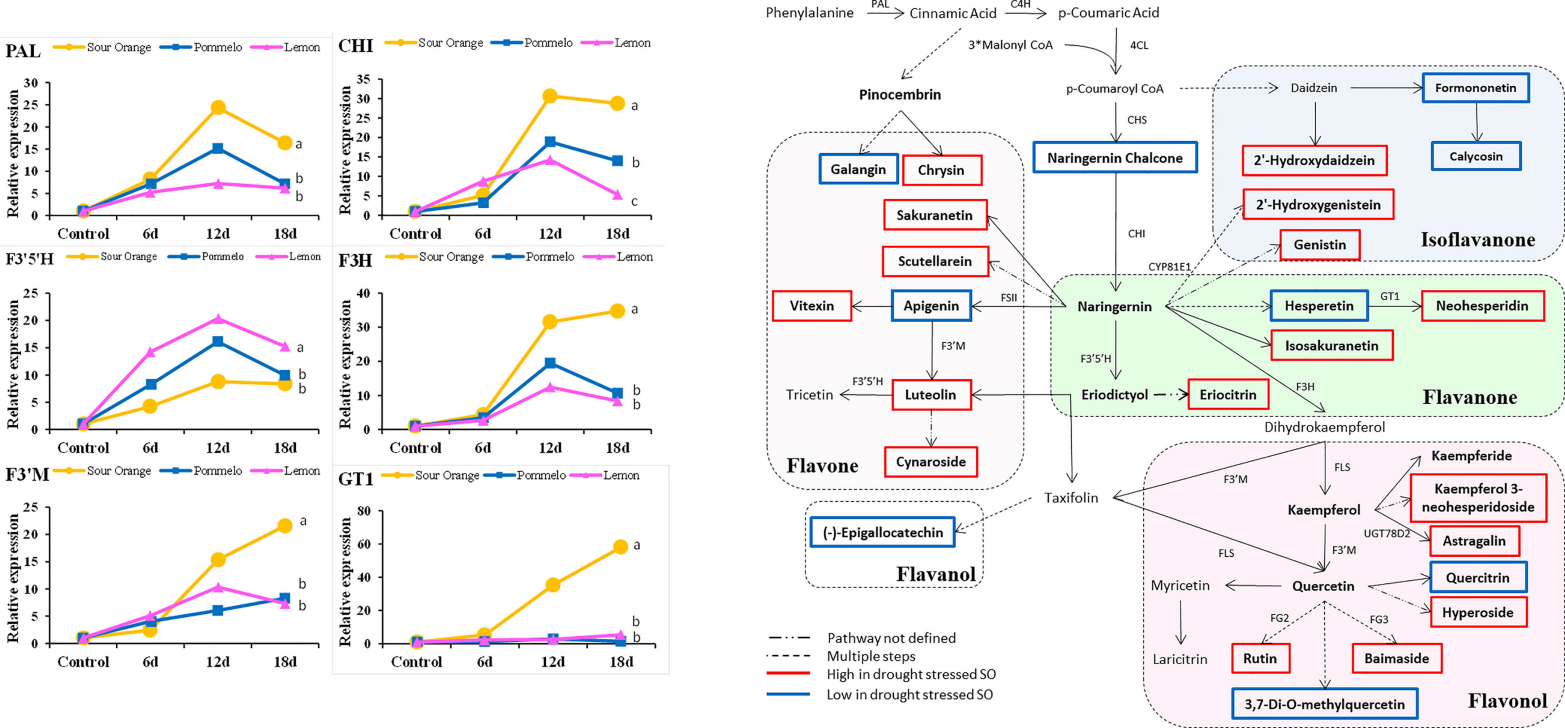
Gene expression analyses of flavonoid pathway genes and flavonoid biosynthesis pathway highlight different flavonoid compounds that were altered under drought stress, particularly in drought-tolerant SO. Abbreviations: SO, sour orange; *PAL*, phenylalanine ammonia-lyase; *CHI*, chalcone isomerase; *F3H*, flavanone 3-hydroxylase; GT1, flavanone 7-O-glucoside 2′′-O-beta-L-rhamnosyltransferase; *F3′5′H*, flavonoid 3′,5′-hydroxylase; *F3*′*M*, flavonoid 3′-monooxygenase. A least significant difference was used to compare the citrus species at p < 0.05 (a–c).

A total of 15 flavonoid biosynthesis pathway genes were selected for examination of their expression level in the drought-stressed citrus species (**Figure 5**). The gene expressions of *PAL*, *CHI*, *F3*′*5*′*H*, *F3H*, *F3*′*M*, and *GT1* are represented in **Figure 5**, whereas the expression level of *C4H*, *4CL*, *CHS*, *FLS*, *FSII*, *FG2*, *FG3*, *CYP81E1*, and *UGT78D2* are presented in **Supplementary Figure 1**. After 18DDS, the *PAL*, *CHI*, *F3H*, *F3’M*, and *GT1* genes were more highly expressed in sour orange leaves than in pummelo and lemon, whereas the *F3*′*5*′*H* gene showed the highest expression in lemon leaves than in pummelo and sour orange after 18DDS (**Figure 5**). In pummelo, *UGT78D2* gene expression was significantly higher as compared with that in lemon and sour orange leaves after 18DDS (**Supplementary Figure S1**). The gene expression of CHS and FSII genes did not alter significantly among sour orange, pummelo, and lemon (**Supplementary Figure S1**). Our results revealed that the expression of the *GT1* (involved in neohesperidin biosynthesis) gene was significantly higher in SO than in pummelo and lemon (**Figure 5**), and the metabolic data also showed that sour orange leaves have a higher maximum content of neohesperidin than lemon and pummelo (**Figure 3**).

According to the metabolic results, we have made a flavonoid biosynthesis pathway to understand which compound in the flavonoid pathway was increased or decreased in response to drought stress particularly in drought-tolerant SO. In sour orange leaves, seven flavones (luteolin, cynaroside, vitexin, scutellarein, sakuranetin, and chrysin), five flavonols (hyperoside, astragalin, rutin, baimaside, and kaemferol 3-neohesperidoside), four flavanones (isosakuranetin, neohesperidin, eriocitrin, and pinocembrin), and three isoflavanones (2′-hydroxydaidzein, 2′-hydroxygenistein, and genistin) were increased after drought stress (**Figure 5**). Some flavonoids showed a decreasing trend in drought-stressed SO such as two flavones (apigenin and galangin), one flavanol ((-)-epigallocatechin), two flavonol (quercitrin and 3,7-di-o-methylquercetin), two isoflavanone (calycosin and formononetin), one flavanone (hesperetin), and one chalcone (naringenin chalcone) (**Figure 5**). The compounds that were decreased after drought stress in SO may not have a significant role in drought tolerance. Additionally, some precursor flavonoids such as apigenin, hesperetin, and naringenin chalcone were decreased in SO after drought stress; this might be because they were converted into downstream flavonoids (**Figure 5**).

### 3.7 Antioxidant activity and biochemical characteristics of citrus species under drought stress

**Figure 6:**
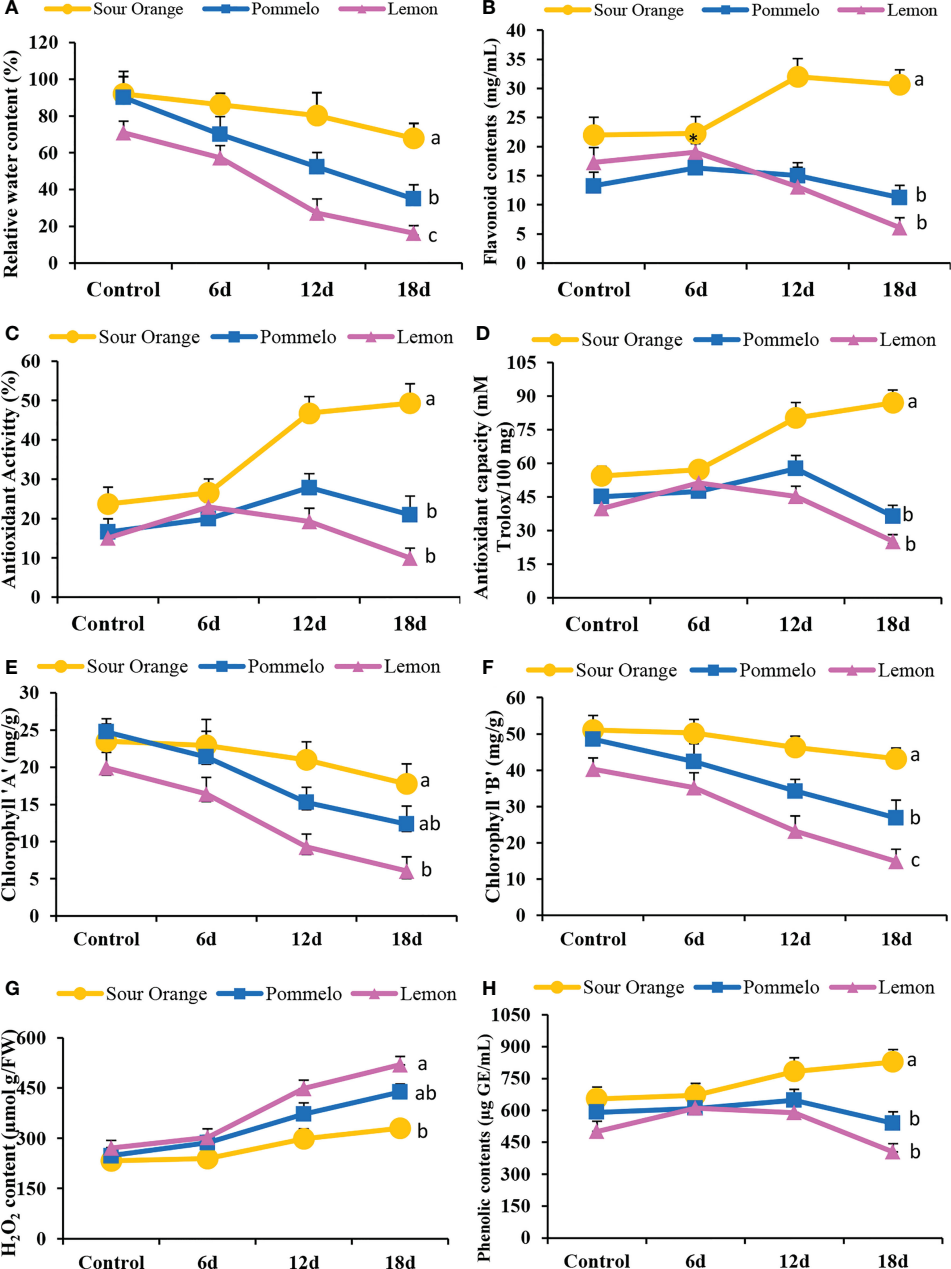
Antioxidant activity and biochemical attributes of citrus leaves under drought stress. **(A)** Relative water content (%), **(B)** total flavonoid content, **(C)** antioxidant activity (%), **(D)** antioxidant capacity, **(E)** chlorophyll a content, **(F)** chlorophyll b content, **(G)** H_2_O_2_ content, **(H)** total phenolic contents. FW, fresh weight; 6d, 6 days of drought stress; 12d, 12 days of drought stress; 18d, 18 days of drought stress. Student’s t-test was used to compare the drought stress intervals and their control samples at p<0.05 (a,b,c).

Drought stress significantly altered the antioxidant activity and biochemical attributes of citrus leaves under drought stress (**Figure 6**). The drought-sensitive lemon showed the lowest relative water content (RWC) in the leaves as compared to pummelo and SO (**Figure 6A**). After 18DDS, the sour orange showed 67.93% RWC, whereas pummelo and lemon showed significantly lower RWCs of 34.89% and 16.19%, respectively (**Figure 6A**). In SO, the flavonoids contents have no obvious difference at 6DDS than its control; however, the lemon and pummelo flavonoids contents increased by 19.05 and 16.35 mg/ml after 6DDS, which is higher than their control by 17.29 mg/ml and 13.24 mg/ml, respectively (**Figure 6B**). After 18DDS, the flavonoid content significantly increased by 30.65 mg/ml in SO as compared with pummelo with 11.22 mg/ml and lemon by 6.06 mg/ml (**Figure 6B**). Similar trends were observed in antioxidant activity and antioxidant capacity (**Figures 6C, D**). After 18DDS, the sour orange significantly increased the antioxidant activity (49.32%) and capacity (86.98 mM Trolox/100 mg), and these were higher than the lemon antioxidant activity (9.94%) and capacity (25.19 mM Trolox/100 mg) (**Figures 6C, D**). These outcomes suggested that the lemon variety failed to tolerate prolonged drought stress, while the sour orange enhanced the flavonoids biosynthesis and antioxidant activity and capacity and tolerated the 18 days of prolonged drought stress.

Chlorophyll a and b contents were altered significantly under drought stress in citrus species (**Figures 6E, F**). After 12DDS and 18DDS, the lemon showed 9.27 and 6.29 mg/g of chlorophyll, respectively, whereas the SO showed 20.98 and 17.76 mg/g, correspondingly (**Figure 6E**). In the case of chlorophyll b, the sour orange showed higher chlorophyll b contents (46.26 and 43.16 mg/g) than lemon (23.23 and 14.91 mg/g) and pummelo (34.27 and 26.89 mg/g) after 12DDS and 18DDS, respectively (**Figure 6F**). The H_2_O_2_ content increased significantly after drought stress in SO, pummelo, and lemon (**Figure 6G**). In sour orange, the H_2_O_2_ content was (329.77 µmol g/FW) significantly lower than pummelo (438.39 µmol g/FW) and lemon (519.9 µmol g/FW), after 18DDS (**Figure 6G**). After 18DDS, the lemon variety showed lower chlorophyll contents and higher H_2_O_2_ content than control lemon plants, which suggests that lemon is highly susceptible to drought stress. In SO, the H_2_O_2_ content was significantly lower than in pummelo and lemon and has significantly higher phenolic and flavonoids contents with maximum antioxidant activity and capacity than pummelo and lemon, after 18DDS (**Figures 6B–D, G, H**), which indicated that sour orange significantly tolerated the drought stress with better antioxidant capacity and increased flavonoid contents compared to lemon and pummelo species.

## 4 Discussion

The genus *Citrus* contains more than 162 species (Swingle, 1967) that possess uneven and diverse levels of secondary metabolites (Zhu et al., 2020). These metabolites provide a secondary defense and contribute to the normal growth and development of plants and help plants to communicate with their surrounding environment (Singh et al., 2020). Recent studies revealed that the citrus species (*Carrizo citrange* and *Citrus latipes*) that have a high level of total metabolites can scavenge the ROS more efficiently during abiotic and biotic stress (Zandalinas et al., 2017b; Rao et al., 2018; Rao et al., 2020). Generally, the cultivated citrus varieties such as lemon, pummelo, sweet orange, and mandarins are more prone to ROS damage (produced during stress), and these varieties possess low levels of total metabolites (Hijaz et al., 2016; Rao et al., 2018). Our results indicated that the drought-tolerant sour orange has a high level of flavonoid compounds, phenolic contents, antioxidant activity, and capacity under drought stress as compared to drought-susceptible lemon and pummelo (**Figures 2**, **3**, **6**).

In citrus species, flavonoids biosynthesis has been increasingly stimulated after several biotic (antibacterial, antifungal, antiviral, etc.) (Rao et al., 2018) and abiotic (metal toxicity, drought, wounding, high light stress, chilling, salt stress, absorption of harmful radiations, and nutrient deficiency) stresses (Pietta, 2000; Zandalinas et al., 2017b; Rao et al., 2019; Rao et al., 2020). Transgenic *Arabidopsis* (overexpression of citrus *CsCYT75B1* gene) stimulates the flavonoid biosynthesis genes such as *PAL*, *C4H*, *4CL*, *CHI*, *F3H*, *F3′M*, and *FLS* under drought stress and accumulated higher total flavonoid contents than the wild type (WT); all the transgenic lines acclimatize the drought stress by neutralizing the ROS than WT (Zandalinas et al., 2017b; Rao et al., 2020). Our qPCR results showed that sour orange leaves have significantly higher expression of *PAL*, *CHI*, *FLS*, *GT1*, *F3H*, *F3’M*, *C4H*, *4CL*, *FG2*, *FG3*, and *CYP81E1* genes than pummelo and lemon, after 18DDS (**Figure 5**, **Supplementary Figure S1**). Previously, it has been reported that flavonoids biosynthesis genes such as *PAL*, *CHS*, *CHI*, *FLS*, *DFR*, and *F3H* (Mohammad et al., 2018) were significantly expressed in citrus species under drought stress (Zandalinas et al., 2017b). The upregulation of these structural genes stimulates the biosynthesis of antioxidant flavonoids such as flavanone, flavones, and flavonols (Zandalinas et al., 2017b). Our metabolic and gene expression results confirmed that the sour orange activates its flavonoid biosynthesis pathway to accumulate fortified antioxidant flavonoids to acclimatize the ROS produced under drought stress, thus maintaining the normal homeostasis between ROS generation and scavenging in SO compared to in lemon and pummelo (**Figures 3**, **5**, **6**).

Flavonoids are localized in plant vacuoles that possess powerful antioxidant properties (Pietta, 2000). The high antioxidant activity of flavonoids is due to the presence of a catechol structure that can scavenge the ROS (produce during stress) (Bors et al., 1990; Pietta, 2000). Flavonoids provide basal defense against a variety of plant pathogens and are involved in seeds dispersal, attract pollination, antiviral, antibacterial, antifungal, defense against insects/pests, and scavengers of ROS (during oxidative stress) (Pourcel et al., 2007). The citrus species that have a high level of endogenous flavonoids can tolerate the citrus Huanglongbing disease and water-deficit condition (Zandalinas et al., 2017b). Some phenolic compounds such as anthocyanins and proanthocyanidins also exhibit decent antioxidant activities, and they play a significant role in protecting the subcellular organelles from abiotic and biotic stresses (Khoo et al., 2017; Meena et al., 2017; Rao et al., 2019; Multari et al., 2020; Rao et al., 2021b). It has been reported that flavonoids significantly increased after several biotic (antibacterial, antifungal, antiviral, etc.) and abiotic (metal toxicity, drought, wounding, high light stress, chilling, salt stress, absorption of harmful radiations, and nutrient deficiency) stresses in different citrus species (Pietta, 2000; Zandalinas et al., 2017b; Rao et al., 2018; Rao et al., 2019; Rao et al., 2020). Our results showed that sour orange biosynthesized specialized flavonoids (such as eriocitrin, genistin, 2′-hydroxydaidzein, 2′-hydroxygenistein, neohesperidin, cynaroside, kaempferol 3-neohesperidoside, chrysin, vitexin, and luteolin) under drought stress, whereas in lemon and pummelo, these flavonoids decreased or had no significant change (**Figures 2**, **3**; **Supplementary Table S2**).

Eriocitrin significantly reduced oxidative stress, promoted cell proliferation, and inhibited cell apoptosis and necrosis (He et al., 2020). Eriocitrin stimulates the superoxide dismutase enzyme (antioxidant defense) activity to acclimatize the stress in rats (He et al., 2020). Several isoflavones such as genistin, daidzin, genistein, and daidzein were increased in soybean roots after osmotic stress, cold stress, and combined osmotic and cold stress (Swigonska et al., 2014). Moderate salt and cold stresses significantly accumulate antioxidant flavonoids such as cynaroside and luteolin in the leaves of two *Capsicum* cultivars (Genzel et al., 2021). *Zanthoxylum bungeanum* (Maxim.) from the family Rutaceae rapidly biosynthesized two flavone compounds such as isovitexin and vitexin in response to drought stress (Hu et al., 2021). In *Scutellaria baicalensis* roots, chrysin was accumulated in response to drought stress, and a high concentration of chrysin helps plants to eliminate the excessive ROS produced during drought stress and maintains the equilibrium in plant cells (Meng et al., 2020). Our results showed that cynaroside, eriocitrin, 2′-hydroxygenistein, 2′-hydroxydaidzein, chrysin, neohesperidin, kaempferol 3-neohesperidoside, baimaside, hyperoside, quercitrin, and vitexin were uniquely higher in the leaves of sour orange after drought stress, whereas these compounds were significantly lower or had no obvious difference in pummelo and lemon under drought stress (**Figure 2**; **Table 2**). The high concentration of aforementioned flavonoids in drought-tolerant SO and the low concentration of these flavonoids in drought susceptible lemons revealed that these flavonoids play a significant role in minimizing the negative effects of drought stress and assisting sour orange with adapting to the water-deficit condition.

Interestingly, an increasing trend of genistin, eriocitrin, and luteolin flavonoids was observed in sour orange during 12DDS and 18DDS, whereas the opposite trend was observed in lemon plants (**Figure 3**). As the intensity of drought stress increases, the concentration of genistin, eriocitrin, and luteolin flavonoids decreased significantly in 12DDS and 18DDS lemon leaves (**Figure 3**). These outcomes showed that a high concentration and increasing trend of specific flavonoids under drought stress in sour oranges would assist SO to withstand prolonged drought stress by rapidly quenching the free radicals, whereas the decreasing trend of specific aforementioned flavonoids with higher ROS (H_2_O_2_) disrupts the normal redox balance in lemon under drought stress.

The highest concentration of hyperoside was observed in sour orange leaves after 12DDS and 18DDS (209.40 and 368.01 µg/g), whereas pummelo and lemon showed no significant change in hyperoside at 12DDS and 18DDS (**Figure 3**; **Supplementary Table S2**). Interestingly after 18DDS, the sour orange leaves have 1,407.43- and 37.6-fold higher concentrations of neohesperidin than pummelo and lemon leaves, respectively; similarly, the kaempferol 3-neohesperidoside was 23.34- and 16.96-fold higher in sour orange than in pummelo and lemon, correspondingly, after 18DDS (**Table 3**). These results suggest that the pummelo and lemon did not have enough antioxidant flavonoids or failed to biosynthesize fortified aforementioned flavonoid compounds, probably due to weak or delayed (different genetic makeup than sour orange) biosynthesis of secondary metabolites, which disturbs the internal ROS production and scavenging homeostasis. Previous literature reported that these three flavonoids (hyperoside, neohesperidin, and kaempferol 3-neohesperidoside) have promising free radical scavenging activity (Borges Bubols et al., 2013; Rao et al., 2021a). Besides their antioxidant activity, these compounds also have pharmacological properties such as neuroprotective, anti-inflammatory, anti-depressant, anti-diabetic, cardio-protective, anti-fungal, radio-protective, anti-cancer, and gastro-protective (Raza et al., 2017). In **Table 2**, several flavonoids were increased and decreased in response to drought stress in SO, pummelo, and lemon. Some compounds (such as neohesperidin, cynaroside, hyperoside, genistin, luteolin, and kaempferol 3-neohesperidoside) were increased in sour orange (drought tolerant) and decreased in lemon (drought susceptible), while some flavonoids such as narcissin, kaempferide, and pinocembrin were increased in lemon and decreased or unchanged in sour orange in response to drought stress (**Table 2**). These results showed that drought tolerance in citrus is a complex metabolic process, dependent on the individual flavonoid compounds and also on the citrus species ability to biosynthesize these specific flavonoid compounds. In the future, an integrated transcriptomic and metabolic study should be conducted to evaluate the metabolic pathways and their correlated genes and do some functional verification of candidate genes associated with flavonoid biosynthesis to understand and improve the drought tolerance in cultivated citrus.

## 5 Conclusions

The current study quantified 37 flavonoid compounds by using LC-MS/MS from three citrus species (sour orange, pummelo, and lemon) and evaluated their response to drought stress. Several flavonoid compounds such as eriocitrin, neohesperidin, cynaroside, hyperoside, genistin, luteolin, and kaempferol 3-neohesperidoside were increased significantly in drought-tolerant sour orange leaves in response to drought stress, whereas these compounds decreased or showed no significant change in drought susceptible lemon leaves. These results showed that lemon plants failed to biosynthesize the specialized flavonoid compounds in response to drought stress. Interestingly, at 18DDS, the neohesperidin was 1,407- and 37-fold higher in sour orange leaves as compared to 18DDS pummelo and lemon leaves, respectively. Moreover, the antioxidant activity and capacity were also increased in SO with higher total contents of phenolics and flavonoids. We concluded that drought tolerance in citrus species is highly correlated with high concentration and increasing trend of specialized flavonoid compounds and also the capability of citrus species to rapidly biosynthesize the aforementioned flavonoid compounds in response to drought stress.

## Data availability statement

The original contributions presented in the study are included in the article/**Supplementary Material**. Further inquiries can be directed to the corresponding authors.

## Author contributions

Conceptualization, SH, MT and MR. Methodology, MR. Software, MR. Validation, BF and MR. Formal analysis, MR. Investigation, MR. Resources, MR, MA and MHA. Data curation, MR, QA, MK and MHA. Writing—original draft preparation, MR. Writing—review and editing, MR, RZ, CM, SH, MT, QX and LW. Visualization, RZ, BF, CM and MR. Supervision, LW. Project administration, MR. Funding acquisition, BF, CM and LW. All authors contributed to the article and approved the submitted version.
