# Supplementary material for: LC-MS/MS-based metabolomics approach identified novel antioxidant flavonoids associated with drought tolerance in citrus species
Source: Front Plant Sci. 2023 Aug 10;14:1150854. doi: 10.3389/fpls.2023.1150854 (PMC10450343; doi:10.3389/fpls.2023.1150854)
Supplement: Supplementary file 1 [file DataSheet_1.docx]

**Flavonoids qualitative analysis and quantification formula:**

**UPLC Conditions**

“The sample extracts were analyzed using an UPLC-ESI-MS/MS system (UPLC， ExionLC™ AD，https://sciex.com.cn/ ; MS，Applied Biosystems 6500 Triple Quadrupole, https://sciex.com.cn/ ). The analytical conditions were as follows, UPLC: column, Waters ACQUITY UPLC HSS T3 C18 (100 mm×2.1 mm i.d.，1.8 µm); solvent system, water with 0.05% formic acid (A), acetonitrile with 0.05% formic acid (B); The gradient elution program was set as follows: 0-1 min, 10%-20% B; 1-9 min, 20%-70% B; 9-12.5 min, 70%-95% B,12.5-13.5 min, 95% B; 13.5-13.6 min, 95%-10% B,13.6-15 min, 10% B; The flow rate was set at 0.35 mL/min and the temperature was set at 40°C. The injection volume is 2 μL.”

**ESI-MS/MS Conditions**

“Linear ion trap (LIT) and triple quadrupole (QQQ) scans were acquired on a triple quadrupole-linear ion trap mass spectrometer (QTRAP), QTRAP® 6500+ LC-MS/MS System, equipped with an ESI Turbo Ion-Spray interface, operating in positive and negative ion mode and controlled by Analyst 1.6.3 software (Sciex). The ESI source operation parameters were as follows: ion source, ESI+/-; source temperature 550 ℃; ion spray voltage (IS) 5500 V (Positive), -4500 V (Negative); curtain gas (CUR) was set at 35 psi, respectively. Flavonoids were analyzed using scheduled multiple reaction monitoring (MRM). Data acquisitions were performed using Analyst 1.6.3 software (Sciex). Multiquant 3.0.3 software (Sciex) was used to quantify all metabolites. Mass spectrometer parameters including the declustering potentials (DP) and collision energies (CE) for individual MRM transitions were done with further DP and CE optimization. A specific set of MRM transitions were monitored for each period according to the metabolites eluted within this period.”

Qualitative analysis of 37 flavonoids was accomplished by comparison of the accurate precursor ions (Q1), product ion (Q3) values, fragmentation patterns, and the retention time (RT) with those obtained by injecting standards using the same conditions, since the standards were available (Sigma-Aldrich, St. Louis, MO, USA; http://www.sigmaaldrich.com/united-states.html).

The integral peak area ratio of all detected citrus samples is substituted into the linear equation of the standard curve, for calculation. After further substitution into the calculation formula, the absolute content data of the substance in the actual sample is finally obtained, as described earlier (Chen et al., 2013). Note: the calculation formula has been converted into units. The sample size can be obtained by directly substituting the corresponding value.

Flavonoid contents in the sample (nmol/g) = C × V / 1000000 / W.

C: Sample concentration value obtained by substituting the integral peak area ratio of the sample into the standard curve (nmol/L)

W: Weighed sample mass (g)

V: Solution volume used for extraction (µL)

**Supplementary Table 1:** Signifies the detail of individual flavonoid compound, calibration curve (standard equation), coefficient of correlation, and minimum- and maximum limits of quantification used in this study.

| **Serial No** | **Compound names** | **KEGG compound ID** | **kegg_map** | **CAS number** | **Equation^a^** | **Coefficient of correlation** | **LLOQ** |
| --- | --- | --- | --- | --- | --- | --- | --- |
| **1** | Naringenin chalcone | C06561 | ko01100,ko01110 | 25515-46-2 | y = 16856.71 x + 1386.35 | 0.99 | 5 |
| **2** | (-)-Epigallocatechin | C12136 | ko00941,ko01110 | 970-74-1 | y = 3591.02 x + 4181.99 | 0.99 | 5 |
| **3** | Neohesperidin | C09806 | ko00941 | 13241-33-3 | y = 5.52 x - 16385.37 | 0.99 | 1 |
| **4** | Isosakuranetin | C05334 | ko00941,ko01110 | 480-43-3 | y = 12920.81 x + 271.67 | 0.99 | 1 |
| **5** | Hesperetin | C01709 | ko00941 | 520-33-2 | y = 3.45 x + 1900.30 | 0.99 | 0.5 |
| **6** | Pinocembrin | C09827 | ko00941 | 480-39-7 | y = 4479.68 x + 1063.09 | 0.99 | 1 |
| **7** | Eriocitrin | C09732 | - | 13463-28-0 | y = 17532.89 x - 1799.49 | 0.99 | 1 |
| **8** | Eriodictyol | C05631 | ko01100,ko01110 | 552-58-9 | y = 4550.56 x - 1734.30 | 0.99 | 5 |
| **9** | Taxifolin | C01617 | ko00941,ko01110 | 480-18-2 | y = 6293.82 x - 22503.41 | 0.99 | 5 |
| **10** | Vitexin | C01460 | ko00941,ko00944 | 3681-93-4 | y = 5.69 x - 12486.38 | 0.99 | 1 |
| **11** | Luteolin | C01514 | ko00941,ko00944 | 491-70-3 | y = 18298.50 x + 3741.68 | 0.99 | 5 |
| **12** | Sakuranetin | C09833 | ko00941,ko01110 | 2957-21-3 | y = 4.27 x + 2830.89 | 0.99 | 1 |
| **13** | Diosmin | C10039 | - | 520-27-4 | y = 3.70 x - 4780.78 | 0.99 | 1 |
| **14** | Apigenin | C01477 | ko00941,ko00944 | 520-36-5 | y = 12713.88 x - 756.19 | 0.99 | 5 |
| **15** | Galangin | C10044 | ko00941 | 548-83-4 | y = 1762.75 x + 444.83 | 0.99 | 10 |
| **16** | Narcissin | - | - | 604-80-8 | y = 24285.22 x - 5030.49 | 0.99 | 5 |
| **17** | Tricetin | C10192 | ko00941 | 520-31-0 | y = 3.59 x - 8072.66 | 0.99 | 5 |
| **18** | Cynaroside | - | - | 1268798 | y = 25915.27 x + 2751.53 | 0.99 | 5 |
| **19** | Acacetin | C01470 | ko00944 | 480-44-4 | y = 5.55 x + 3.031e | 0.99 | 5 |
| **20** | Scutellarein | C10184 | - | 529-53-3 | y = 4277.33 x + 11537.65 | 0.99 | 5 |
| **21** | Limocitrin | - | - | 489-33-8 | y = 2306.05 x - 3624.52 | 0.99 | 10 |
| **22** | Chrysin | C10028 | ko00941 | 480-40-0 | y = 6722.57 x - 32.11 | 0.99 | 5 |
| **23** | Baimaside | C12667 | ko00944,ko01110 | 18609-17-1 | y = 3.01 x - 9294.51 | 0.99 | 1 |
| **24** | Astragalin | C12249 | ko00944,ko01110 | 480-10-4 | y = 26569.61 x + 5206.16 | 0.99 | 1 |
| **25** | 3,7-Di-O-methylquercetin | C01265 | ko00944,ko01110 | 61395 | y = 1.35 x + 5.77e | 0.99 | 1 |
| **26** | Rutin | C05625 | ko00944,ko01110 | 153-18-4 | y = 4.01 x - 11812.44 | 0.99 | 1 |
| **27** | Quercitrin | C01750 | ko00944 | 522-12-3 | y = 17884.29 x - 7175.78 | 0.99 | 1 |
| **28** | Kaempferide | C10098 | ko00944 | 491-54-3 | y = 9.92 x + 6.654e | 0.99 | 1 |
| **29** | Kaempferol 3-neohesperidoside | - | - | 32602-81-6 | y = 17028.06 x - 2318.09 | 0.99 | 5 |
| **30** | Laricitrin | C12633 | ko00944,ko01110 | 53472-37-0 | y = 18391.03 x + 3925.67 | 0.99 | 5 |
| **31** | Myricetin | C10107 | ko00941,ko01110 | 529-44-2 | y = 16340.26 x - 3.95e | 0.99 | 5 |
| **32** | Hyperoside | - | - | 482-36-0 | y = 38.79 x + 1611.92 | 0.99 | 1 |
| **33** | 2'-Hydroxygenistein | C12134 | ko00943 | 1156-78-1 | y = 27073.69 x + 7065.48 | 0.99 | 1 |
| **34** | 2'-Hydroxydaidzein | C02495 | ko00943,ko01110 | 7678-85-5 | y = 21986.67 x + 9881.96 | 0.99 | 5 |
| **35** | Genistin | - | - | 529-59-9 | y = 2886.73 x + 11091.65 | 0.99 | 10 |
| **36** | Formononetin | C00858 | ko00943,ko01110 | 485-72-3 | y = 1.11 x + 3533.76 | 0.99 | 1 |
| **37** | Calycosin | C01562 | ko00943,ko01110 | 20575-57-9 | y = 4.64 x - 56.06 | 0.99 | 1 |

a: the calibration curve; ULOQ: Upper Limit of Quantification is 2000 for all compounds; LLOQ: Lower Limit of Quantification

12-DS: 12 days of drought stress; 18-DS: 18 days of drought stress; SO: sour orange.

**Supplementary figure 1:** Gene expression analyses of flavonoid pathway genes. Abbreviations: Cinnamate 4-hydroxylase (*C4H*), 4-coumarate CoA ligase (*4CL*), Chalcone synthase (*CHS*), Flavonol synthase (*FLS*), Flavone synthase II (FSII), Flavonol-3-O-glucoside L-rhamnosyltransferase (FG2), Flavonol-3-O-glucoside/galactoside glucosyltransferase (FG3), Cytochrome P450 81E8-like (CYP81E1), UDP-GLUCOSYL TRANSFERASE 78D2 (UGT78D2). Least significant difference was used to compare the citrus varieties at p < 0.05 (a,b,c).

.

**References:**

Chen, W., Gong, L., Guo, Z., Wang, W., Zhang, H., Liu, X., Yu, S., Xiong, L., Luo, J., 2013. A novel integrated method for large-scale detection, identification, and quantification of widely targeted metabolites: application in the study of rice metabolomics. Mol. Plant 6, 1769–1780.
